# Evaluation of Aroma Characteristics of Dried Shrimp (*Litopenaeus vannamei*) Prepared by Five Different Procedures

**DOI:** 10.3390/foods11213532

**Published:** 2022-11-06

**Authors:** Weizhen Sun, Hongwu Ji, Di Zhang, Zewei Zhang, Shucheng Liu, Wenkui Song

**Affiliations:** 1 Guangdong Provincial Key Laboratory of Aquatic Product Processing and Safety, College of Food Science and Technology, Guangdong Ocean University, Zhanjiang 524088, China; 2 Guangdong Provincial Engineering Technology Research Center of Seafood, College of Food Science and Technology, Guangdong Ocean University, Zhanjiang 524088, China; 3 Guangdong Province Engineering Laboratory for Marine Biological Products, College of Food Science and Technology, Guangdong Ocean University, Zhanjiang 524088, China; 4 Key Laboratory of Advanced Processing of Aquatic Product of Guangdong Higher Education Institution, College of Food Science and Technology, Guangdong Ocean University, Zhanjiang 524088, China; 5 Collaborative Innovation Center of Seafood Deep Processing, Dalian Polytechnic University, Dalian 116034, China

**Keywords:** dried shrimps, aroma characteristics, volatile compounds, sensory evaluation, aroma-active compounds (AACs), signature aroma compounds, drying method

## Abstract

*Litopenaeus vannamei* is one of the most popular shrimp species in the world and has been reported in studies on its dryness and flavor. However, the aroma characteristics of shrimps dried with different drying methods are compared in a unified way, and there are few reports on the difference in aroma of different shrimps dried. In order to clarify the difference in aroma characteristics of shrimp dried produced by different drying methods. In this study, blanched shrimp (BS) was used as a control to analyze the aroma characteristics of shrimp dried by five different procedures (SD-BFDP) samples, namely vacuum freeze-dried shrimp (VFDS), vacuum dried-shrimp (VDS), heat pump-dried shrimp (HPDS), hot air dried-shrimp (HADS) and microwave vacuum-dried shrimp (MVDS). An electronic nose (E-nose) was used to obtain the aroma fingerprint of SD-BFDP samples. Headspace solid-phase microextraction gas chromatography-mass spectrometry (HS-SPME-GC-MS) was used for qualitative and quantitative analysis of volatile compounds in SD-BFDP samples. Partial least squares regression (PLSR) was used to analyze potential correlations between sensory attributes and aroma-active compounds (AACs). Partial least squares-discrimination analysis (PLS-DA) was used to screen for signature aroma compounds. The results of the E-nose showed that there were differences in the aroma fingerprints of the SD-BFDP samples, and the E-nose could distinguish the five kinds of SD-BFDP. The qualitative and quantitative results of GC-MS showed that the types and contents of the main volatile components of SD-BFDP samples were different. 15 AACs were screened from SD-BFDP based on odor activity value (OAV). The PLSR results showed good correlations between certain sensory attributes and the majority of AACs. PLS-DA results displayed that aroma attributes of SD-BFDP samples could be distinguished by six signature aroma compounds, including trimethylamine, 2,5-dimethylpyrazine, 2-ethyl-5-methylpyrazine, nonanal, 3-ethyl-2,5-dimethylpyrazine, and octanal. These research results reveal that shrimps dried in different procedures have unique aroma characteristics, which could provide a theoretical basis for the rapid identification of aroma attributes of dried shrimps in the future. From a flavor perspective, MVD is the best drying method.

## 1. Introduction

*Litopenaeus vannamei* is one of the most widely farmed shrimp species in China and one of the major aquatic products in the world. It is favored by consumers due to its delicious meat and rich nutrition. After the fresh shrimp was dried, the storage period of the product could be extended, and the transportation cost of the product could be effectively reduced [1]. Because of its unique flavor and bright color, it was a great willingness by consumers and has been used in instant soup, fast food, and baby food.

Different drying methods have been widely used in the processing of aquatic products, and there are certain reports on the research on its flavor characteristics. Some studies showed that the contents of aldehydes, ketones, and alcohols were lower in VFDS and cold-air-dried fillets, while the levels of aldehydes, ketones, and alcohols were higher in HADS and VDS fillets. The reason may be that the levels of these compounds were inhibited in the low-temperature drying process [2,3]. Meanwhile, some of the literature reported that the high temperature often promoted the formation of other volatile flavor compounds. With increasing in HPDS temperature from 5 °C to 35 °C, the contents of alcohols, aldehydes, ketones, and acid were increased, especially aliphatic and aromatic hydrocarbons in HPDS-dried squid [4,5]. In addition, there were more kinds and contents of volatile compounds in microwave-dried shrimps. It may be that the microwave had a greater impact on the lipid distribution of shrimp [6]. The amount of esters in the dried pufferfish with MVD is higher than that of HAD, which may be the decomposition of heat-sensitive compounds caused by the high temperature of HAD [7].

In recent years, the methods used to analyze the flavor of food mainly include E-nose, gas chromatography-olfactory-mass spectrometer (GC-O-MS), headspace-gas chromatography-ion mobility spectrometry (GC-IMS), and GC-MS. The E-nose system is composed of a series of sensors and identification systems. The E-nose is convenient for preprocessing, has good repeatability and high sensitivity, and has been widely used to distinguish odor differences between different samples [1,6]. However, the E-nose system mainly analyzes the overall information of the sample and cannot obtain detailed information on volatile compounds. GC-O-MS consists of two working units, GC-O and GC-MS, which combine the features of the two devices into an integrated instrument. Faster and more accurate analysis of key odorants by GC-O-MS, avoiding false detection of odorants [8]. However, GC-O-MS pretreatment requires a lot of repetitive and time-consuming work, such as aroma extract dilution analysis [9]. Therefore, it is not suitable for the rapid characterization of volatile compounds in food. GC-IMS combines the high separation power of GC with the fast response of IMS [9]. It has been widely used in the analysis of volatile components in food [10,11]. Because the response of IMS is nonlinear, GC-IMS has limitations in accurate quantitative analysis and lacks databases such as the NIST mass spectral library [9]. GC-MS has the advantages of high sensitivity and high resolution and is widely used in the qualitative and quantitative analysis of volatile components in food [8,12]. Jaffres et al. studied the quality changes of boiled and peeled shrimp during storage in modified atmosphere packaging and analyzed the volatile components in shrimp samples by GC-MS [13]. It can be seen that a single analytical method cannot provide comprehensive information about the flavor profile.

In order to understand the effect of different drying methods on the aroma characteristics of dried shrimp, insight into the differences in aroma characteristics of dried shrimps in different processes. In this study, E-nose and SPME-GC-MS were used for the differential analysis of aroma characteristics in shrimp dried by five different procedures (VFDS, VDS, HPDS, HADS, and MVDS). PLS-DA was used to screen out the signature aroma compounds that distinguish the aroma characteristics of shrimp dried by five different procedures. The results of these techniques can be used to analyze the aroma characteristics of different shrimps dried while providing basic data for the rapid identification and differentiation of dried shrimps in the future.

## 2. Materials and Methods

### 2.1. Samples and Reagents

*L. vannamei*, with an average weight of 13.0 ± 1.0 g, was purchased from Huguang Market, Zhanjiang City, (Guangdong, China), stored in a foam box with ice and shipped to the laboratory within 1 h, and immediately stored at −40 °C refrigerator.

Nonanoic acid methylester (chromatographically pure, purity ≥99.9%) was purchased from Shanghai Macklin Biochemical Co., Ltd. (Shanghai, China). The mixed standard of n-alkanes (C_5_~C_32_) was purchased from Shanghai Anpel Experimental Technology Co., Ltd. (Shanghai, China).

### 2.2. Sample Preparation

The fresh shrimp were blanched in brine at 100 °C (salt concentration: 3.0%, shrimp-water mass ratio of 1:3) for 3 min, then removed the shrimp and drained, and the water content of the shrimp was measured to be 72.28 ± 0.04%.

The blanched shrimp (BS) was put into a drying box for drying to obtain a dried shrimp with a moisture content of 20%. The drying conditions refer to previous studies with slight modifications, as follows:

(1) VFDS: was pre-frozen at −40 °C for 12 h and then placed in a vacuum freeze dryer (FDU-1100, Tokyo Rika, Japan) at −50 °C under a drying chamber pressure of 20 Pa for 16 h, with a loading capacity of 5308 g/m^2^. Refer to the method of Sun et al. [14] with a slight modification.
(2) VDS: was performed for 17 h in a vacuum drying oven (VOS-201SD, Tokyo Rika, Japan) at 50 °C and a pressure of 0.07 Mpa, with a loading capacity of 4 kg/m^2^. Refer to the method of He [15] with a slight modification.
(3) HPDS: was performed in a heat pump dryer (L3.5TB1, Wilson, Guangdong, China) with a temperature of 30 °C and a wind speed of 2.0 m/s for 25 h, with a loading capacity of 6 kg/m^2.^ Refer to the method of Shi, Xue, Zhao, Li, and Wang [16] with a slight modification.
(4) HADS: was performed in a hot air-drying oven (DHG-9023A, Heheng, Shanghai, China) with a temperature of 80 °C and a wind speed of 1.0 m/s for 6 h, with a loading capacity of 1587 g/m^2^. Refer to the method of Sun et al. [14] with a slight modification.
(5) VMDS: was performed in a microwave vacuum drying oven (RWBZ-08S, Sunray, Nanjing, China) with a power of 400 W and a vacuum of 0.08 Mpa for 20 min, with a loading capacity of 75 g/33 dm^3^. Refer to the method of Duan [17] with a slight modification.

### 2.3. E-Nose Analysis

The experimental method is referenced from the literature by Hu, Wang, Liu, Cao, and Xue, with slight modifications [18]. The overall aroma profile of dried shrimps was detected via the E-nose system with the PEN3 (AirSense Analytics GmbH, Schwerin, Germany). There were 10 metal oxide sensors in the PEN3 system, and each sensor had different sensitivities to chemical substances (Table 1). 2.0 g of dried shrimps’ powder was weighed and placed into a 25 mL headspace bottle and sealed with a silicone stopper. The sample was equilibrated at 60 °C for 5 min and then was measured. All the samples were repeated 3 times. The measurement time of the E-nose signal was 100 s, the cleaning time was 120 s, and the headspace gas was pumped into the sensor array volume at a constant rate of 400 mL/min.

### 2.4. SPME-GC–MS Analysis of Volatile Compounds

SPME was used for the analysis of volatile compounds in dried shrimp samples. The method was slightly modified by Zhang, Ji, Liu, and Gao [19]. An accurately weighed 2.0 g of dried shrimps’ powder was placed into a headspace bottle, and 2 μL of methyl pelargonate (145.83 ng/g in methanol) was added as an internal standard. Then, the headspace bottle was placed on a constant temperature water bath, and inserting the preconditioned SPME fibers (50/30 μm DVB/CAR /PDMS solid phase microextraction, Supelco, Bellefonte, PA, USA) into the headspace bottle were extracted at 75 °C for 35 min.

GC-MS analysis was performed on a Shimadzu TQ8050NX gas chromatography-mass spectrometer (Shimadzu, Kyoto, Japan) equipped with an InertCap^®^ Pure-WAX quartz capillary column (30 m × 0.25 mm, 0.25 μm, Shimadzu, Kyoto, Japan). The initial oven temperature was maintained at 40 °C for 3 min, then raised to 100 °C at 4 °C/min, maintained for 2 min; and then raised to 230 °C at 8 °C/min, maintained for 5 min. He (purity ≥99.999%) was the carrier gas at 1 mL/min. The electron ionization energy was 70 eV, the ion source temperature was 230 °C, the mass scanning range was *m*/*z* 33–550, and the acquisition mode was Q3 full scan.

### 2.5. Qualitative and Quantitative Analysis of Volatile Components

Volatile compounds were identified by comparison to standard compounds with mass spectra, Kovats retention indices (RI), and retention times. The RI of volatile compounds was calculated by using the retention time of C5-C32 n-alkane standards (Shanghai Anpel Experimental Technology Co., Shanghai, China) under the same analytical conditions. In addition, compared with published data previously reported in the published literature and listed in several credible online databases (http://www.odour.org.uk, http://www.flavornet.org, accessed on 12 October 2022). Preparation of n-alkanes: Take an appropriate amount of the alkane mixed standard purchased, mix it with methanol and add it to the headspace vial. Other operating conditions are the same as the sample analysis. The content of volatile compounds were calculated using the standard internal method. OAV was calculated as the ratio of the concentration of a volatile component to the threshold value of that compound in water. Compounds with OAV ≥1 were considered to be AACS with a significant impact on the aroma characteristics of dried shrimps. Calculated as follows:

(1) $$\mathrm{RI}=100\times \left(\frac{t_{x\;}-t_{n}}{t_{n+1}+t_{n}}+n\right)$$

(2) $$C_{i}=\frac{A_{i}}{A_{s}\times m_{i}}\times m_{s}$$

(3) $$\mathrm{OAV}=\frac{C_{i}}{T}$$

where: formula (1): t_x_, t_n_, and t_n+1_ represent the retention time of the volatile compounds to be tested, n-carbon atoms n-alkanes and n + 1 carbon atoms n-alkanes, respectively (t_n_ < t_x_ < t_n+1_). n represents the number of carbon atoms; formula (2): C_i_ is the concentration of the compound (ng/g), A_i_ and A_s_ are the peak area of compound i and the peak area of the internal standard, respectively, m_i_ is the mass of the sample (g), m_s_ is the mass of the internal standard (ng); formula (3): T is the threshold for the compound in water [20].

### 2.6. Sensory Analysis

Sensory analysis was performed by the method of Zhang, Ji, Liu, and Gao [19] with slight modifications. The sensory analysis panel was performed by 10 members (5 males and 5 females, aged 20 to 25) from the School of Food Science and Technology, Guangdong Ocean University (Guangdong, China). Each panelist has accumulated extensive experience in food sensory description. Before the analysis, members of the sensory analysis participated in 3 training sessions (Sensory training was scheduled 7 days, 3 days, and 1 day before the formal sensory evaluation, respectively) to become familiar with the sensory description of dried shrimps. Before the sensory evaluation, the panelists were forbidden to eat for at least 1 h. Six sensory descriptors were selected to evaluate the sensory characteristics of the samples, including smoky (benzaldehyde); sweet-flavor (maltol); roasted-flavor (2,5-dimethylpyrazine); cooked-meat-like (3-(methylthio) propanal); caramel (4-hydroxy-2,5-dimethyl-3(2H)-furanone); and fishy (trimethylamine).

Each sample weighed 5.0 g and transferred into a 20 mL headspace bottle marked with a random three-digit code. After the headspace bottle was incubated in a 60 °C water bath for 10 min, each sample was evaluated in triplicate by members of the sensory analysis. Each evaluation was limited to 5 min. In order to avoid feeling fatigued, there was a 5-min rest interval between the two repetitions. The entire evaluation process is limited to 30 min. The whole process of the sensory evaluation experiment was completed in the standard sensory laboratory (temperature 25 °C of the School of Food Science and Technology. For aroma profile evaluation, the intensity of each aroma quality is ranked on a scale from 0 (not perceptible) to 5 (very intense).

### 2.7. Statistical Analysis

All experiments were repeated 3 times, starting from the preparation of dried shrimps, and each sample test was repeated more than 3 times, and the results were expressed in the form of mean value ± standard deviation. SPSS 26 software (IBM, Armonk, NY, USA) was used for the experimental data analysis, and one-way ANOVA was performed to test for significant differences (*p* < 0.05, significant difference). Unscrambler X version 10.4 (CAMO ASA, Oslo, Norway) was used for PLSR. SIMCA 14.1 software (Umetrics, Umea, Sweden) was used for PLS-DA analysis. Origin 2022b software (Origin Lab Inc., Northampton, MA, USA) was used for the development of radar and other graphs.

## 3. Results and Discussion

### 3.1. E-Nose Analysis

**Figure 1:**
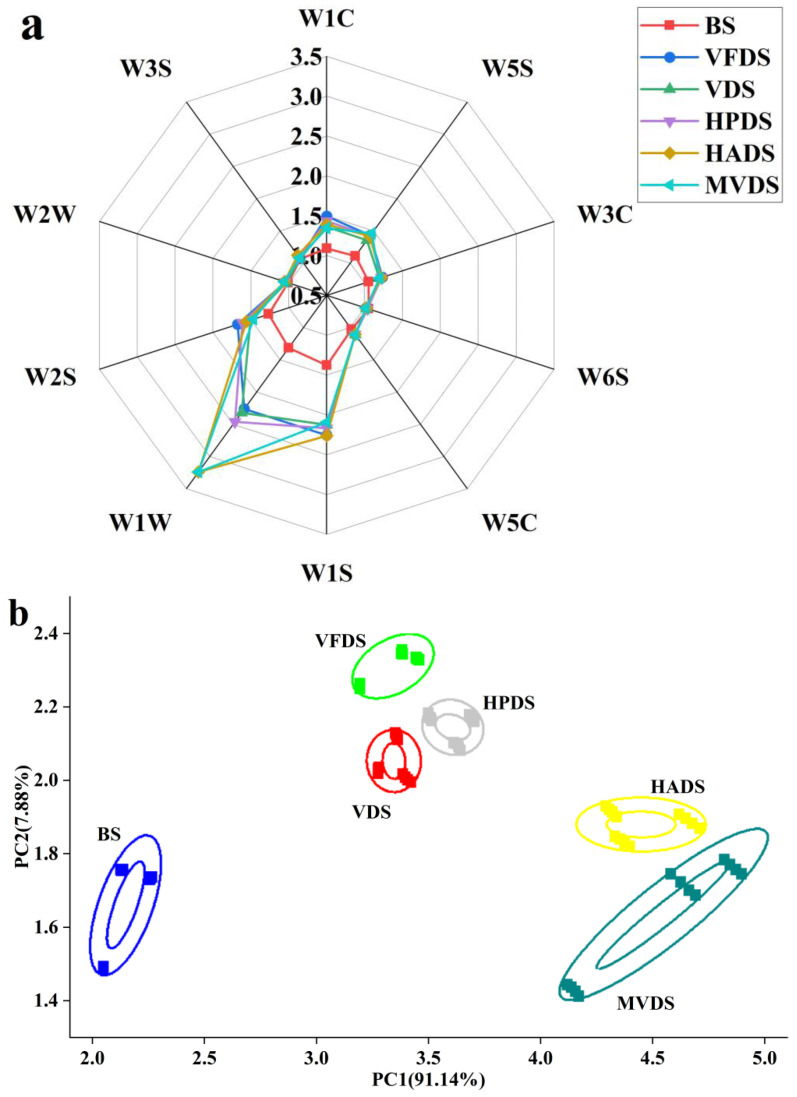
E-nose radar map of dried shrimps’ samples with different drying methods (**a**), PCA diagram of dried shrimps’ samples with different drying methods (**b**). BS: blanching shrimp; VFDS: freeze-dried vacuum shrimp; VDS: vacuum-dried shrimp; HPDS: heat pump-dried shrimp; HADS: hot air-dried shrimp; MVDS: microwave vacuum-dried shrimp.

The E-nose system is a fast, simple, and reproducible bionic detection instrument that can determine the overall aroma profile of a sample. Slight changes in the content of volatile compounds in the sample may lead to different sensor responses [21]. As shown in Figure 1a, the response value of the dried shrimp’s sensor was significantly larger than that of BS, indicating that the overall aroma of the dried shrimps was significantly improved. The sensor response values were different between SD-BFDP samples, indicating that the aroma characteristics of the SD-BFDP samples were different. The higher the E-nose sensor response, the more volatile compounds were in the sample. The highest sensor response value was W1W, followed by W1S, W2S, and W5S, indicating that the SD-BFDP samples contained more sulfides, pyrazines, alcohols, aldehydes, ketones, and compounds with methyl groups. In addition, the aroma fingerprint of HADS and MVDS were similar, whereas the aroma fingerprint of VFDS, VDS, and HPDS was similar. However, the response peak intensity of HADS and MVDS at the sensor W1W was significantly higher than that of VFDS, VDS, and HPDS, indicating that the content of sulfide in the two dried shrimps was higher. This may be due to the fact that sulfide-containing compounds were affected by temperature as Maillard reaction products [22].

Principal component analysis (PCA) was used to classify the aroma characteristics of SD-BFDP samples. The larger the cumulative contribution rate in PCA, the better it can reflect the overall information of each sample. As shown in Figure 1b, the contribution rates of PC1 and PC2 were 91.14% and 7.88%, respectively, and the total contribution rate was 99.02%, indicating that the two principal components could reflect the information from the original data. The SD-BFDP samples were far away from the BS, which indicated that the drying had greatly changed the volatile components of the shrimp. The SD-BFDP samples had different distributions in the PCA space, and all of them could be completely separated, indicating that PCA could well distinguish the SD-BFDP samples. In addition, the SD-BFDP samples were spatially divided into two regions, where VFDS, VDS, and HPDS were one region, and HADS and MVDS were another region. The dried shrimps’ samples in the region were close to each other, indicating that the volatile components were similar to a certain extent, and the volatile components of dried shrimps between regions were quite different. This difference may be attributed to the difference in temperature of the five drying methods, which affects the formation of alcohols, aldehydes, ketones, and acids during the drying process [5].

### 3.2. Volatile Components Analyses

To further determine the effect of different drying methods on the types and contents of volatile components in dried shrimps, GC-MS was used to analyze the volatile components of SD-BFDP samples. As shown in Table S1, a total of 96 volatile compounds were identified in all samples, including aldehydes (15), ketones (10), alcohols (11), esters (6), Pyrazines (19), hydrocarbons (19), acids (7), amines (4) and other heterocyclic compounds (5). In samples with different drying methods, the following numbers of volatile components were identified: 38 (BS), 50 (VFDS), 47 (VDS), 54 (HPDS), 67 (HADS) and 60 (MVDS). In samples with different drying methods, the total contents were 62.63 153.93, 236.36, 280.52, 409.78, and 342.23 ng/g, respectively. It could be seen that there were obvious differences in the types and contents of volatile components in SD-BFDP samples. This may be due to different cooking conditions such as heat transfer mechanism, heating time, and temperature [23]. The types and contents of volatile compounds in BS were the lowest, but the types and contents of BS were significantly increased after drying. It was demonstrated that dried could promote the formation of volatile compounds. The content of volatile components of VFDS in SD-BFDP samples was the lowest, probably because volatile compounds such as aldehydes, esters, heterocycles, acids, and other volatile compounds in frozen samples are more likely to be lost by sublimation under vacuum conditions [24].

**Figure 2:**
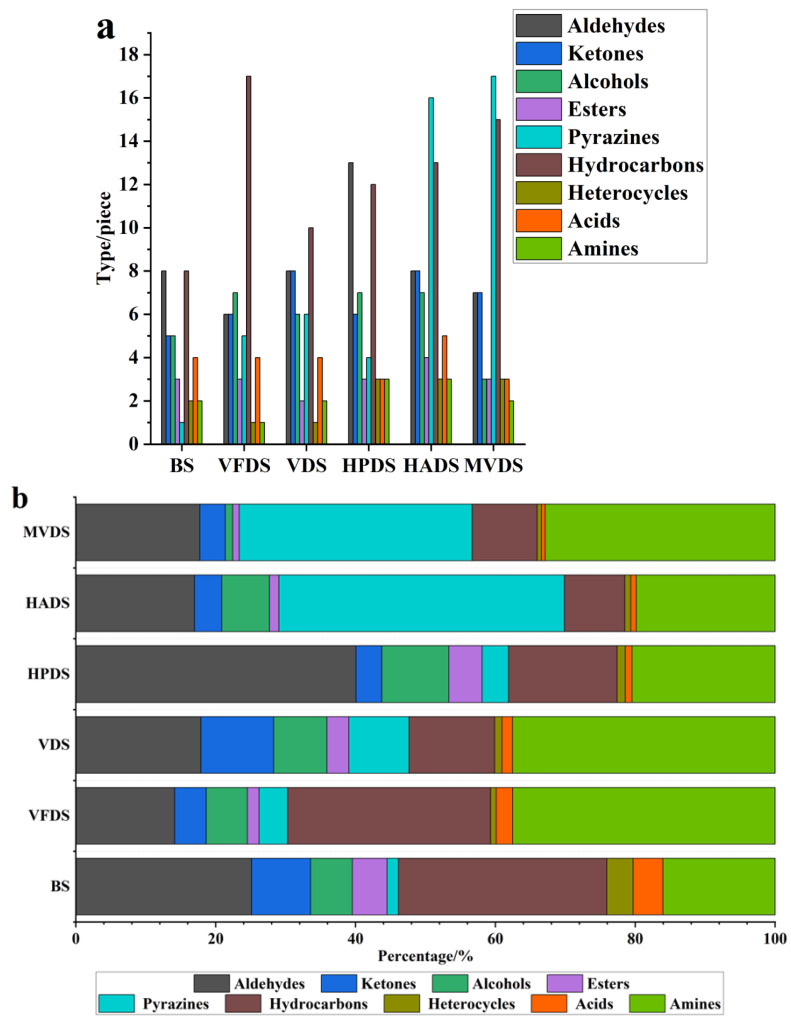
Comparison of volatile compounds in dried shrimps’ samples with different drying methods (**a**), Comparison of relative contents of volatile components in dried shrimp samples with different drying methods (**b**). BS: blanching shrimp; VFDS: freeze-dried vacuum shrimp; VDS: vacuum-dried shrimp; HPDS: heat pump-dried shrimp; HADS: hot air-dried shrimp; MVDS: microwave vacuum-dried shrimp.

As shown in Figure 2a, compared with BS, the dried shrimps had significantly more hydrocarbon species, and VFDS had the most hydrocarbon species. HPDS has the most types of aldehydes; HADS and MVDS were the most types of pyrazines. As shown in Figure 2b, compared with BS, SD-BFDP samples have a significantly lower proportion of hydrocarbons except for VFDS, indicating that low-temperature conditions are conducive to the formation of hydrocarbon compounds. However, hydrocarbons contribute less to flavor due to their high odor threshold [25]. The highest content in VFDS and VDS is amines, accounting for 37.54 % and 37.55 % of the total content, respectively. The most important one is trimethylamine, which occupies a high proportion of SD-BFDP samples. The study by Liang et al. [26]. also showed that trimethylamine formation could be observed during processing, and trimethylamine was detected in each sample after heat treatment. This is caused by the thermal decomposition of trimethylamine oxide [17]. HPDS has the highest aldehyde content, accounting for 40.05% of the total, followed by BS, accounting for 25.12% of the total. In other studies, cooked shrimp were thought to have the highest levels of aldehydes [27]. This result shows that the HPD procedure is easy to make shrimps to generate aldehydes. HADS and MVDS had the highest content of pyrazine, accounting for 40.83% and 33.28% of the total, respectively. In other studies, roasted shrimp had the highest pyrazine concentration, followed by shrimp with a microwave drying procedure [27]. This may be due to the difference in drying temperature, and high-temperature conditions are favorable for the production of pyrazine species [28]. The study by Liang et al. [26] showed that the amount of nitrogen-containing compounds in shrimp samples increased rapidly after heat treatment.

### 3.3. OAV Analysis

Based on the concentrations and thresholds of the aforementioned volatile components, their contribution to the overall aroma was determined by calculating the OAV (Table 2). OAV ≥ 1 indicates that the volatile component contributes to the odor and could be considered AACS. In this study, 15 odorant-active compounds were detected, including six aldehydes, five pyrazines, two alcohols, one ketone, and one amine. In addition, four AACS were detected in BS, indicating that the shrimp had less odor after cooking, while the OAV of volatile shrimp compounds changed significantly after drying. In dried shrimps, the following quantities of AACS were identified VFDS(6), VDS(11), HPDS(12), HADS(12), and MVDS(11).

Aldehydes are important volatile components in food. The aldehydes of C5-C9 are mainly produced by the oxidation and degradation of fat. Due to the low threshold of aldehydes, they have a great contribution to the flavor of meat products [29]. 3-Methylbutanal could impart the aroma of food, chocolate, and coffee. In addition to no contribution to VFDS. 3-Methylbutanal also contributes to the other four dried shrimps, especially contributing more to the aroma of HADS and MVDS. Nonanal has rose odors, which is the characteristic scent of VFDS, VDS, and HPDS, and HPDS has the highest OAV (31.14). (E,Z)-2,6-nonadienal was the characteristic aroma of HPDS, giving food waxy, grassy odor. (E,Z)-2,6-nonadienal was one of the AACs in surimi [30]. Hexanal makes food have a fragrant taste, and octanal has a strong fruity odor, which has a certain contributes to the aroma of dried shrimp. According to previous studies, aldehydes were considered to be the main contributors to the aroma of cooking seafood, and hexanal was considered to have a greater contribution to the overall aroma characteristics of steamed crabs [31]. Octanal was considered to be one of the key aroma sources in dry-cured fish [32].

Ketones are divided into short-chain ketones and long-chain ketones, which may be produced by the thermal oxidation of polyunsaturated fatty acids or amino acid degradation and have a unique aroma and fruity aroma [33]. 2-Nonanone contributes to the aroma of VDS and MVDS, which could impart creamy and fruity flavors to food.

Alcohols were usually produced by the action of lipoxygenase on fatty acids or by the reduction of carbonyl compounds [34]. In this study, octanol contributed to the aroma of five kinds of dried shrimp, with a clear aroma and a sweet taste. 1-Octen-3-ol belonged to aliphatic unsaturated alcohols. Except for no contribution to MVDS, 1-octen-3-ol also contributed to the aroma of the other four dried shrimps, with mushroom and fishy smells.

Amines such as trimethylamine are used to determine the freshness of aquatic products with fishy and ammonia odors [35]. The OAV of trimethylamine in the SD-BFDP samples was larger, and trimethylamine contributed the most to the VFDS, which may be one of the reasons for its poor flavor. The increase in trimethylamine during heating may be due to the thermal decomposition of choline, betaine, methionine, or trimethylamine oxide [36].

Pyrazines and other N-containing heterocyclic compounds are important volatile compounds in dried shrimps [19]. Pyrazine is mainly generated through the interaction between Strecker-degraded amines and alpha-dicarbonyls and has odor characteristics such as meaty, roasted-flavor, nutty, coffee, roasted potato, and popcorn [37]. 2,5-Dimethylpyrazine, 3-ethyl-2,5-dimethylpyrazine, and 2-ethyl-5-methylpyrazine had higher OAV in dried shrimps, indicating that the three compounds contributed more to the aroma of dried shrimps. The highest OAV in HADS and MVDS was 2-ethyl-5-methylpyrazine, 315.91, 280.34, respectively, followed by 3-ethyl-2,5-dimethylpyrazine, 74.56, 70.26, respectively. Therefore, the flavors of HADS and MVDS were similar, which was consistent with the results of the E-nose, and also proves that pyrazine compounds have a great contribution to the flavor of dried shrimps.

### 3.4. Sensory Evaluation

**Figure 3:**
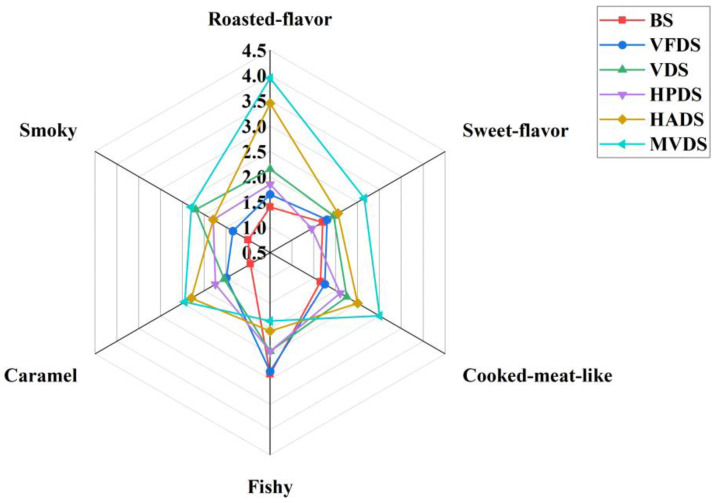
Sensory evaluation of flavor characteristics of different dried shrimps. BS: blanching shrimp; VFDS: vacuum freeze-dried shrimp; VDS: vacuum-dried shrimp; HPDS: heat pump-dried shrimp; HADS: hot air-dried shrimp; MVDS: microwave vacuum-dried shrimp.

To more intuitively show the flavor differences among the SD-BFDP samples, sensory evaluation was used to analyze the SD-BFDP samples. As shown in Figure 3, compared with BS, the fishy smell score of dried shrimps were decreased, and the other flavor scores were improved to varying degrees. This result indicated that the dried shrimp had a better flavor. This was due to the Maillard reaction producing large amounts of volatile compounds such as pyrazines, aldehydes, ketones, furans, and sulfide-containing thermal processing [38]. Compared with the other four dried shrimps, MVDS had the highest score in roasted-flavor, smoky, caramel, cooked-meat-like, sweet-flavor, and the lowest score in fishy, so MVDS had the best flavor. The HADS score profile was similar to MVDS, but the fishy score was higher than that of MVDS, and the scores of other flavors were slightly lower than MVDS, so the HADS flavor was second to MVDS. The aroma characteristics of HPDS, VDS, and VFDS were similar, and there was no significant difference. However, VDS scores higher in roasted flavor and smoky, so VDS flavor is better than HPDS and VFDS. Among the three dried shrimps, although VFDS had the highest sweet-flavor score, the fishy score was also the highest, which affected the overall flavor of VFDS. The overall aroma of VFDS was weak, so the flavor was the worst. The sensory evaluation results were consistent with those of the E-nose and GC-MS.

### 3.5. Multivariate Statistical Analysis

#### 3.5.1. Correlation between Sensory Attributes of Different Dried Shrimps and AACs

**Figure 4:**
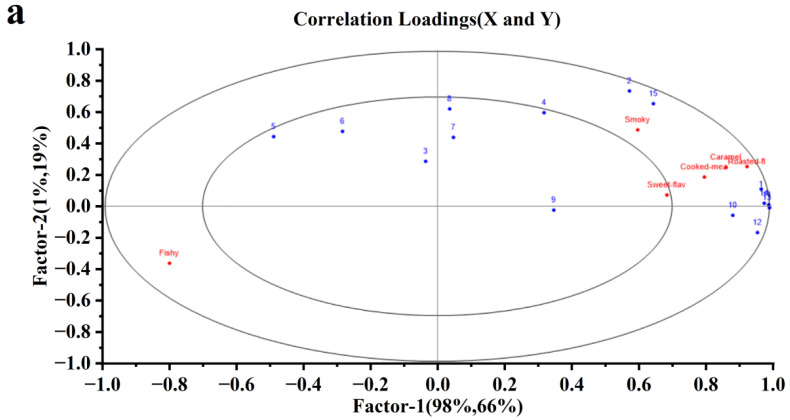

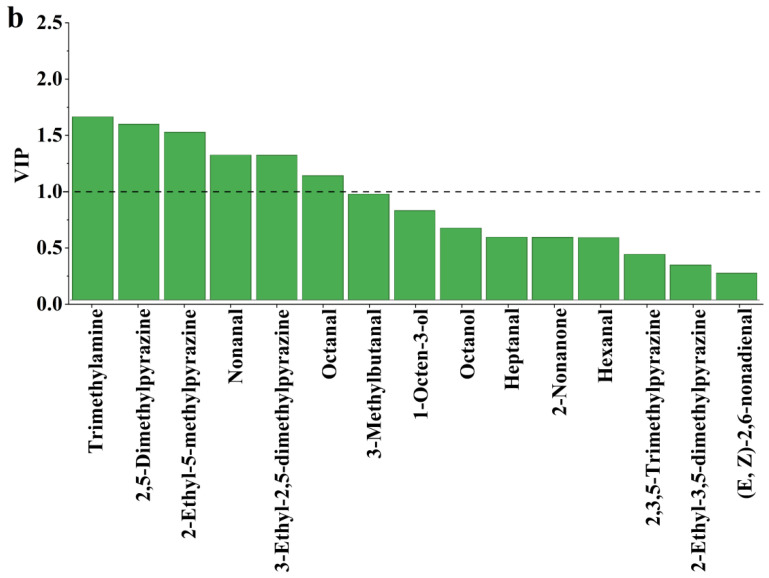
PLSR correlation loadings plot of different dried shrimp AACS and sensory attributes (**a**), 1: 3-Methylbutanal; 2: Hexanal: 3: Heptanal: 4: Octanal: 5: Nonanal; 6: (E, Z)-2,6-nonadienal; 7: 2-Nonanone; 8: Octanol; 9: 1-Octen-3-ol; 10: 2,5-Dimethylpyrazine; 11: 3-Ethyl-2,5-dimethylpyrazine; 12: 2,3,5-Trimethylpyrazine; 13: 2-Ethyl-5-methylpyrazine; 14: 2-Ethyl-3,5-dimethylpyrazine;15: Trimethylamine.VIP ranking chart of different dried shrimp AACS (**b**). BS: blanching shrimp; VFDS: freeze-dried vacuum shrimp; VDS: vacuum-dried shrimp; HPDS: heat pump-dried shrimp; HADS: hot air-dried shrimp; MVDS: microwave vacuum-dried shrimp.

In order to understand the correlation between sensory attributes of different dried shrimps and AACs, PLSR was used to analyze the relationship between sensory attributes an AACs, PLSR has been widely used in correlative sensory and GC datasets [39]. As shown in Figure 4a, AACs were designated as the independent variable, and six sensory attributes (smoky, sweet-flavor, roasted-flavor, cooked-meat-like, caramel, fishy) were designated as dependent variables. The derived PLSR model between the AACs and sensory attribute matrices explained 99% and 85% of the variance in X (AACs) and Y (sensory attributes), respectively. Except for heptanal, octanal, nonanal, (E, Z)-2,6-nonadienal, 2-nonanone, octanol, 1-octen-3-ol, the rest of the AACs and sensory attributes were located between the small and large ellipses. The results showed that AACs between two ellipses could be considered to correlate with sensory attributes, while the seven AACs within the small ellipse were poorly correlated. Hexanal and trimethylamine were positively correlated with smoky. 3-methylbutanal, 2,5-dimethylpyrazine, 3-ethyl-2,5-dimethylpyrazine, 2,3,5-trimethylpyrazine, 2-ethyl-5-methylpyrazine, 2-ethyl-3,5-dimethylpyrazine were positively correlated with caramel, cooked-meat-like and roasted-flavor. PLSR results were consistent with sensory assessment and AACs results.

#### 3.5.2. PLS-DA of Different Shrimp Dried Odor Active Compounds

In order to identify the signature aroma compounds of different dried shrimps, PLS-DA was used for further analysis of AACs in SD-BFDP samples. PLS-DA is a supervised discriminant analysis statistical method which can effectively interpret the observed values and realize the prediction of the corresponding variables. The permutation test of 200 responses was used to verify the PLS-DA model, and the obtained R^2^ = 0.239, Q^2^ = −0.690, The intercept of Q^2^ on the Y axis was a negative value, indicating that this model has no overfitting phenomenon and could be used for subsequent determination of marker volatile compounds.

The contribution of each variable to the sample was quantified according to the variable importance in the project (VIP) in the PLS-DA model, and the volatile components with VIP > 1 were called signature aroma compounds. In previous studies, volatile compounds with VIP > 1 were considered to be the key aroma compounds of sugar-smoked chicken legs [40]. The larger the VIP, the more significant the difference in the content of this volatile component among the SD-BFDP samples, which means that different drying methods have a greater impact on the volatile components of dried shrimp. As shown in Figure 4b, six AACs with VIP > 1 were identified, namely trimethylamine, 2,5-dimethylpyrazine, 2-ethyl-5-methylpyrazine, nonanal, 3-ethyl-2,5-dimethylpyrazine, and octanal, which could be used as signature aroma compounds to distinguish five different dried shrimps. Trimethylamine and 2,5-dimethylpyrazine were also considered to be the signature flavor compounds of stored shrimp heads [41].

## 4. Conclusions

In this study, E-nose, GC-MS, and sensory analysis were used to analyze aroma characteristics differences of SD-BFDP samples. Results showed that drying methods had a significant effect on the aroma characteristics of SD-BFDP samples. The types and contents of their main volatile components exhibited significant differences. The PLSR analysis showed good correlations between certain sensory attributes and 15 AACs. Aroma attributes difference of SD-BFDP samples can be distinguished by six signature aroma compounds, including trimethylamine, 2,5-dimethylpyrazine, 2-ethyl-5-methylpyrazine, nonanal, 3-ethyl-2,5-dimethylpyrazine, octanal. In sum, the aroma attributes of SD-BFDP samples were as follows MVDS > HADS > VDS> HPDS > VFDS. These findings will provide a theoretical basis for the rapid identification of aroma attributes of dried shrimps.

## Figures and Tables

**Table 1:** E-nose sensors and their main application in PEN3.

Array Serial No.	Sensor Name	Representative Material Species	Performance Description
1	W1C	Aromatic compounds	Sensitive to aromatic constituents, benzene
2	W5S	Broad range	Sensitive to nitrogen oxides
3	W3C	Aromatic	Sensitive aroma, ammonia
4	W6S	Hydrogen	Sensitive to hydrides
5	W5C	Arom-aliph	Short-chain alkane aromatic component
6	W1S	Broad-methane	Sensitive to toluene
7	W1W	Sulphur-organic	Sensitive to sulfides, terpenes
8	W2S	Broad-alcohol	Sensitive to alcohols, aldehydes and ketones
9	W2W	Sulph-chlor	Sensitive to aromatics, organosulfur compounds
10	W3S	Methane-aliph	Sensitive to long-chain alkanes

**Table 2:** Comparison of AACS content in different dried shrimp samples.

NO.	Compounds Name	Threshold (ng/g) ^1^	CAS	Formula	Odorant Description ^2^	OAV ^3^					
						BS ^4^	VFDS ^4^	VDS ^4^	HPDS ^4^	HADS ^4^	MVDS ^4^
1	3-Methylbutanal	1.1	590-86-3	C_5_H_10_O	chocolate, coffee	ND	ND -	1.10	5.40	23.20	23.91
2	Hexanal	5	66-25-1	C_6_H_12_O	Grassy, Creamy	<1	<1	1.57	1.42	1.31	2.03
3	Heptanal	3	111-71-7	C_7_H_14_O	fishy	<1	ND	<1	2.21	1.24	ND
4	Octanal	0.587	124-13-0	C_8_H_16_O	Fatty, fruity	1.42	2.69	1.74	10.02	4.36	8.15
5	Nonanal	1	124-19-6	C_9_H_18_O	Rose, fat	5.77	3.80	7.26	31.14	ND	ND
6	(E, Z)-2,6-nonadienal	0.8	557-48-2	C_9_H_14_O	waxy, grassy	ND	ND	ND	22.70	ND	ND
7	2-Nonanone	5	821-55-6	C_9_H_18_O	creamy, fruity	<1	ND	2.29	ND	<1	1.13
8	Octanol	1	111-87-5	C_8_H_18_O	Fragrance, sweet	<1	<1	1.72	6.75	2.12	2.87
9	1-Octen-3-ol	1.5	3391-86-4	C_8_H_16_O	Mushroom, fishy	<1	1.23	3.56	6.94	10.47	ND
10	2,5-Dimethylpyrazine	0.8	123-32-0	C_6_H_8_N_2_	Nutty, roasted	1.27	3.90	10.46	9.18	38.53	18.17
11	3-Ethyl-2,5-dimethylpyrazine	0.4	13360-65-1	C_8_H_12_N_2_	roasted, smoky	ND	2.38	6.55	4.80	74.56	70.26
12	2,3,5-Trimethylpyrazine	11	14667-55-1	C_7_H_10_N_2_	Nutty, Caramel	ND	ND	ND	ND	4.07	2.32
13	2-Ethyl-5-methylpyrazine	0.04	13360-64-0	C_7_H_10_N_2_	smoky, burnt	ND	ND	26.54	19.57	315.91	280.34
14	2-Ethyl-3,5-dimethylpyrazine	2.2	13925-07-0	C_8_H_12_N_2_	roasted aroma	ND	ND	ND	ND	7.26	<1
15	Trimethylamine	2.4	75-50-3	C_3_H_9_N	fishy, ammonia	3.84	24.08	36.82	23.55	33.52	46.82

^1^ Reference reported thresholds. ^2^ Reports in References. ^3^ ND: not detected. The ratio of Ci and T. Ci is the concentration of the compound (ng/g), T is the threshold for the compound in water. ^4^ BS: blanching shrimp; VFDS: freeze-dried vacuum shrimp; VDS: vacuum-dried shrimp; HPDS: heat pump-dried shrimp; HADS: hot air-dried shrimp; MVDS: microwave vacuum-dried shrimp.

## Data Availability

The data used to support the findings of this study can be made available by the corresponding author upon request.

## Supplementary Materials

The following supporting information can be downloaded at: https://www.mdpi.com/article/10.3390/foods11213532/s1, Table S1: GC-MS analysis of samples with different drying methods. Reference [42] is cited in the supplementary materials.

## Abbreviations

**Acronyms/Abbreviations**	**Full Scientific Name**
BS	Blanched shrimp
SD-BFDP	Shrimp dried by five different procedure
VFDS	Vacuum freeze dried shrimp
VDS	Vacuum dried shrimp
HPDS	Heat pump dried shrimp
HADS	Hot air dried shrimp
MVDS	Microwave vacuum dried shrimp
E-nose	Electronic nose
HS-SPME-GC-MS	Headspace solid-phase microextraction gas chromatography-mass spectrometry
AACs	Aroma active compounds
PLSR	Partial least squares regression
PLS-DA	Partial least squares-discrimination analysis
OAV	Odor activity value
