# Supplementary material for: Evaluation of Aroma Characteristics of Dried Shrimp (Litopenaeus vannamei) Prepared by Five Different Procedures
Source: Foods. 2022 Nov 6;11(21):3532. doi: 10.3390/foods11213532 (PMC9658951; doi:10.3390/foods11213532)
Supplement: Supplementary file 1 [file foods-11-03532-s001.zip › foods-1962101-supplementary.pdf]

Table S1. GC-MS analysis of samples with different drying methods.

Volatile components	RI[1]	ID[2]	CAS	Formula	Odour description[3]	Concentration /(ng/g)[4]					
						**BS[5,6]**	**VFDS[5,6]**	**VDS[5,6]**	**HPDS[5,6]**	**HADS[5,6]**	**MVDS[5,6]**
**Aldehydes**											
Benzaldehyde	1489	MS, RI	100-52-7	C_7H_6O	Bitter, almond	2.78±0.15^{e}	11.45±0.79^{d}	20.50±1.71^{c}	35.01±0.30^{a}	28.80±1.84^{b}	18.93±1.68^{c}
Pentanal	981	MS, RI	110-62-3	$C_5H_{10}O$	Fruity	0.87±0.03^{b}	ND	1.02±0.11^{b}	6.84±0.90^{a}	ND	ND
3-Methylbutanal	936	MS, RI	590-86-3	$C_5H_{10}O$	Chocolate, coffee	ND	ND	1.21±0.15^{b}	5.93±1.16^{b}	25.52±5.87^{a}	26.30±2.08^{a}
Hexanal	1070	MS, RI	66-25-1	$C_6H_{12}O$	Grassy, creamy	2.46±0.02^{c}	3.63±0.59^{c}	7.87±0.38^{b}	7.09±0.09^{b}	6.55±0.25^{b}	10.13±0.91^{a}
Heptanal	1168	MS, RI	111-71-7	$C_7H_{14}O$	Fishy	0.93±0.05^{c}	ND	2.17±0.01^{bc}	6.64±0.34^{a}	3.73±0.91^{b}	ND
Octanal	1278	MS, RI	124-13-0	$C_8H_{16}O$	Fatty, fruity	0.83±0.08^{c}	1.58±0.18^{bc}	1.02±0.06^{c}	5.88±0.01^{a}	2.56±0.01^{b}	4.78±0.88^{a}
Nonanal	1380	MS, RI	124-19-6	$C_9H_{18}O$	Rose, fat	5.77±0.16^{b}	3.80±0.16^{b}	7.26±0.23^{b}	31.14±2.42^{a}	ND	ND
(E, Z)-2,6-nonadienal	1567	MS, RI	557-48-2	$C_9H_{14}O$	Waxy, grassy	ND	ND	ND	1.02±0.07	ND	ND
Decanal	1484	MS, RI	112-31-2	$C_{10}H_{20}O$	Orange peel	1.88±0.52	1.07±0.32	ND	ND	ND	ND
(E)-4-Decenal	1530	MS, RI	65405-70-1	$C_{10}H_{18}O$	Green ,fatty	ND	ND	ND	4.82±0.79	ND	ND
Undecanal	1594	MS	112-44-7	$C_{11}H_{22}O$	Strong fatty	ND	ND	ND	1.90±0.71	ND	ND
Tetradecanal	1924	MS	124-25-4	$C_{14}H_{28}O$	Fatty,fishy	ND	ND	ND	ND	ND	0.30±0.04
Pentadecanal	2023	MS	2765-11-9	$C_{15}H_{30}O$	Fresh waxy	ND	ND	ND	2.25±0.32^{a}	0.34±0.03^{b}	0.11±0.01^{b}
Hexadecanal	2130	MS	629-80-1	$C_{16}H_{32}O$	Cardboard	ND	ND	ND	1.00±0.17	0.51±0.03	ND
cis,cis-7,10,-hexadecadienal	1821	MS	56829-23-3	$C_{16}H_{28}O$	NE	0.20±0.03^{cd}	0.20±0.06^{cd}	1.16±0.18^{bc}	2.84±0.83^{a}	1.42±0.01^{b}	0.14±0.01^{d}
**Ketones**											
Acetophenone	1615	MS	98-86-2	C_8H_8O	Sweet	ND	ND	ND	ND	2.27±0.22	1.90±0.57
2-Heptanone	1171	MS, RI	110-43-0	$C_7H_{14}O$	Musty, peardrops, soapy	1.68±0.17^{b}	3.17±0.07^{ab}	3.66±0.40^{ab}	3.49±0.48^{ab}	5.25±1.12^{a}	ND
6-Methyl-2-heptanone	1226	MS	928-68-7	$C_8H_{16}O$	Camphoreous	ND	ND	ND	ND	0.55±0.20	0.43±0.02
6-Methyl-5-hepten-2-one	1323	MS, RI	110-93-0	$C_8H_{14}O$	Sweet, fruity	0.90±0.08^{a}	0.88±0.02 ^{a}	1.53±0.37 ^{a}	1.40±0.20 ^{a}	ND	ND
2-Octanone	1273	MS, RI	111-13-7	$C_8H_{16}O$	Soapy, floral	ND	1.15±0.14^{ab}	0.54±0.09^{d}	0.87±0.10^{bc}	1.41±0.08^{a}	0.83±0.09^{cd}
2-Nonanone	1377	MS, RI	821-55-6	$C_9H_{18}O$	Creamy, fruity	1.52±0.16^{bc}	ND	11.47±2.65^{a}	1.25±0.08^{c}	1.71±0.22^{bc}	5.66±0.07^{b}
2-Decanone	1479	MS, RI	693-54-9	$C_{10}H_{20}O$	Fruity	ND	ND	0.72±0.25^{c}	ND	3.22±0.17^{a}	1.75±0.18^{b}
2-Undecanone	1589	MS, RI	112-12-9	$C_{11}H_{22}O$	Green, musty	0.20±0.05^{d}	0.46±0.10^{bc}	0.53±0.01^{bc}	1.15±0.08^{a}	0.41±0.04^{cd}	0.66±0.05^{b}
(Z)-6-Undecen-2-one	1612	MS	107853-70-3	$C_{11}H_{20}O$	Rose	ND	0.75±0.17	4.52±0.18	ND	ND	ND
6,10-Dimethyl-5,9-undecadien-2-one	1841	MS	689-67-8	$C_{13}H_{22}O$	Rose ,fruity	0.99^{cd}	0.56±0.07^{d}	1.64±0.04^{b}	2.18±0.35^{a}	1.22±0.08^{bc}	1.18±0.01^{bc}
**Alcohols**											
1-Pentanol	1241	MS	71-41-0	$C_5H_{12}O$	Green; wax	1.12±0.13^{b}	3.21±0.97^{a}	3.95±0.56^{a}	ND	2.36±0.73^{ab}	ND
1-Penten-3-ol	1152	MS, RI	616-25-1	$C_5H_{10}O$	Burnt, meaty	ND	ND	4.24±0.02	ND	1.56±0.04	ND
1-Hexanol	1342	MS	111-27-3	$C_6H_{14}O$	Green, grassy	ND	ND	ND	1.34±0.09	ND	ND
2-Ethyl-1-hexanol	1497	MS	104-76-7	$C_8H_{18}O$	Herbal, carrots	0.79±0.04^{d}	1.27±0.32^{bc}	ND	4.27±0.13^{a}	1.42±0.23^{b}	ND

1-Hexen-3-ol	1148	MS	4798-44-1	$C_6H_{12}O$	Green, fruity, fatty	ND	ND	ND	2.37±0.18	ND	ND
2-Propyl-1-heptanol	1661	MS	10042-59-8	$C_{10}H_{22}O$	NE	ND	ND	ND	ND	2.46±0.52	ND
Octanol	1552	MS, RI	111-87-5	$C_8H_{18}O$	Fragrance, sweet	0.55±0.07^{d}	0.88±0.08^{cd}	1.72±0.18^{bcd}	6.75±1.03^{a}	2.12±0.51^{bc}	2.87±0.02^{b}
1-Octen-3-ol	1438	MS, RI	3391-86-4	$C_8H_{16}O$	Mushroom, fishy	1.03±0.04^{d}	1.85±0.28^{d}	5.34±0.08^{c}	10.41±0.96^{b}	15.71±1.13^{a}	ND
1-Nonanol	1651	MS, RI	143-8-8	$C_9H_{20}O$	Dusty, oily	0.25±0.03^{b}	0.19±0.04^{b}	2.08±0.64^{a}	ND	2.34±0.70^{a}	0.57±0.01^{b}
1-Decanol	1752	MS	112-30-1	$C_{10}H_{22}O$	Sweet,fruity	ND	1.44±0.73	ND	0.83±0.17	ND	ND
1-Undecanol	1873	MS	112-42-5	$C_{11}H_{24}O$	Rose ,fruity	ND	0.22±0.06^{b}	0.72±0.21^{ab}	0.94±0.17^{ab}	ND	0.21±0.02^{b}
**Esters**											
Methyl 2-methyloctanoate	1385	MS	2177-86-8	$C_{10}H_{20}O_2$	NE	1.90±0.34^{a}	2.14±0.57^{a}	2.51±0.92^{a}	ND	ND	2.06±0.05^{a}
Dibutyl phthalate	1971	MS	84-74-2	$C_{16}H_{22}O_4$	Slightly toasted	ND	0.17±0.06^{b}	ND	7.64±0.51^{a}	0.15±0.03^{b}	0.12^{b}
Dimethyl phthalate	2261	MS	131-11-3	$C_{10}H_{10}O_4$	Odorless	ND	ND	ND	0.48±0.04	0.44±0.13	ND
Caproic acid vinyl ester	1315	MS	3050-69-9	$C_8H_{14}O_2$	NE	0.78±0.12^{d}	ND	4.88±0.15^{b}	5.22±0.04^{a}	4.09±0.08^{c}	1.04±0.01^{d}
Triethyl phosphate	1636	MS	78-40-0	$C_6H_{15}O_4P$	Mild cider	ND	ND	ND	ND	0.87±0.06	ND
Hydrogen isocyanate	1190	MS	75-13-8	CHNO	NE	0.44	0.25±0.07	ND	ND	ND	ND
**Pyrazines**											
Pyrazine	1192	MS, RI	290-37-9	$C_4H_4N_2$	Roasted	ND	ND	ND	ND	ND	1.08±0.19
2-Methylpyrazine	1243	MS, RI	109-08-0	$C_5H_6N_2$	Nutty; cacao, roasted	ND	0.47±0.20^{c}	4.82±1.76^{ab}	ND	6.78±0.03^{a}	2.85±0.05^{bc}
2,3-Dimethylpyrazine	1324	MS, RI	5910-89-4	$C_6H_8N_2$	Nutty, coffee,roasted	ND	ND	ND	ND	2.05±0.11	1.82±0.15
2,5-Dimethylpyrazine	1299	MS, RI	123-32-0	$C_6H_8N_2$	Nutty, roasted	1.02±0.08^{d}	3.12±0.40^{d}	8.37±0.20^{c}	7.34±1.03^{c}	30.83±0.77^{a}	14.54±1.42^{b}
2,6-Dimethylpyrazine	1306	MS, RI	108-50-9	$C_6H_8N_2$	Nutty,roasted	ND	1.04±0.15^{c}	2.47±0.17^{b}	ND	5.20±0.20^{a}	2.81±0.62^{b}
2-Ethyl-5-methylpyrazine	1370	MS, RI	13360-64-0	$C_7H_{10}N_2$	Smoky, burnt	ND	ND	1.06±0.07^{b}	0.78±0.14^{b}	12.64±0.68^{a}	11.21±0.18^{a}
2-Ethyl-6-methylpyrazine	1365	MS, RI	13925-03-6	$C_7H_{10}N_2$	Roasted potato	ND	ND	1.00±0.14^{bc}	0.59±0.23^{c}	2.59±0.25^{a}	1.58±0.12^{b}
2,6-Diethylpyrazine	1414	MS	13067-27-1	$C_8H_{12}N_2$	Nutty	ND	ND	ND	ND	0.43±0.07	ND
2-Acetyl-3-methylpyrazine	1595	MS	23787-80-6	$C_7H_8N_2O$	Nutty ,roasted	ND	0.69±0.18^{b}	ND	ND	1.99±0.05^{a}	1.40±0.28^{ab}
2-ethenyl-6-methyl-Pyrazine	1461	MS	13925-09-2	$C_7H_8N_2$	Hazelnut	ND	ND	ND	ND	2.08±0.15	0.97±0.24
3-Ethyl-2,5-dimethylpyrazine	1424	MS, RI	13360-65-1	$C_8H_{12}N_2$	Roasted, smoky	ND	0.95±0.05^{b}	2.62±0.15^{b}	1.92±0.01^{b}	29.83±2.01^{a}	28.11±0.43^{a}
2-Ethyl-3,5-dimethylpyrazine	1439	MS, RI	13925-07-0	$C_8H_{12}N_2$	Roasted aroma	ND	ND	ND	ND	15.96±1.92	17.66±1.42
2-Acetyl-3,5-dimethylpyrazine	1648	MS	54300-08-2	$C_8H_{10}N_2O$	Nutty,roasted,hazelnut	ND	ND	ND	ND	ND	0.16±0.06
3,5-Diethyl-2-methylpyrazine	1474	MS, RI	18138-05-1	$C_9H_{14}N_2$	Nutty,meaty,vegetable	ND	ND	ND	ND	4.63±0.17	3.22±0.27
2,3,5-Trimethylpyrazine	1383	MS, RI	14667-55-1	$C_7H_{10}N_2$	Nutty, caramel	ND	ND	ND	ND	44.72±1.25	25.56±2.41
2,3,5,6-Tetramethylpyrazine	1450	MS	1124-11-4	$C_8H_{12}N_2$	Nutty, musty ,coffee	ND	ND	ND	ND	2.44±0.07	3.73±0.88
2,5-Dimethyl-3-(3-methylbutyl)-pyrazine	1644	MS	18433-98-2	$C_{11}H_{18}N_2$	Fruity	ND	ND	ND	ND	2.40±0.07	2.98±0.25
2,3,5-Trimethyl-6-ethylpyrazine	1494	MS	17398-16-2	$C_9H_{14}N_2$	Nutty	ND	ND	ND	ND	2.75±0.18	ND

2,5-Dimethyl-3-(2-methylpropyl)-pyrazine	1511	MS	32736-94-0	$C_{10}H_{16}N_2$	Nutty	ND	ND	ND	ND	ND	0.45±0.14
**Hydrocarbons**											
Styrene	1238	MS	100-42-5	C_8H_8	Sweet balsam	ND	ND	ND	ND	2.73±0.60	1.97±0.23
3,5,5-Trimethyl-2-hexene	1471	MS	26456-76-8	C_9H_{18}	NE	1.05±0.09^{d}	0.57±0.06^{e}	1.80±0.21^{c}	5.05±0.09^{a}	2.67±0.14^{b}	0.45±0.05^{e}
1,3,5,7-Cyclooctatetraene	1234	MS	629-20-9	C_8H_8	NE	0.43±0.16	8.27±0.73	ND	ND	ND	ND
1-Decene	1036	MS	872-5-9	$C_{10}H_{20}$	NE	ND	1.56±0.16	ND	ND	ND	1.68±0.28
Naphthalene	1701	MS	91-20-3	$C_{10}H_8$	Tar	1.23±0.35^{a}	0.36±0.01^{b}	1.64±0.05^{a}	ND	ND	0.60±0.07^{b}
(3E,5Z)-1,3,5-Undecatriene	1968	MS	19883-29-5	$C_{11}H_{18}$	NE	ND	0.39±0.07^{c}	ND	1.38±0.04^{a}	0.85±0.02^{b}	ND
1-Tridecene	1250	MS	2437-56-1	$C_{13}H_{26}$	NE	ND	0.69^{b}	1.02±0.20^{ab}	1.19±0.07^{a}	ND	ND
a-Farnesene	1724	MS	502-61-4	$C_{15}H_{24}$	Herbal	0.96±0.03^{b}	1.84±0.08^{a}	ND	1.65±0.23^{a}	0.33±0.12^{c}	0.56±0.13^{bc}
Butylated hydroxytoluene	1899	MS	128-37-0	$C_{15}H_{24}O$	Camphor	ND	0.42±0.03^{b}	ND	1.10±0.15^{a}	0.23±0.01^{b}	0.24±0.12^{b}
2,4-Di-tert-butylphenol	2290	MS	96-76-4	$C_{14}H_{22}O$	Waxy floral aroma	ND	ND	ND	1.12±0.09^{a}	0.80±0.08^{a}	0.31±0.12^{b}
Nonylcyclopropane	1250	MS	74663-85-7	$C_{12}H_{24}$	NE	ND	1.09±0.14^{b}	1.44±0.09^{a}	ND	ND	0.37±0.03^{c}
Nonylcyclopentane	1287	MS	2882-98-6	$C_{14}H_{28}$	NE	ND	0.33±0.02^{b}	0.35±0.03^{b}	0.61±0.10^{ab}	0.89±0.27^{a}	ND
3-Methylnonane	980	MS	5911-04-6	$C_{10}H_{22}$	NE	ND	3.18±0.66	ND	ND	ND	4.81±0.01
Undecane	1098	MS	1120-21-4	$C_{11}H_{24}$	NE	11.42±2.89^{a}	5.86±0.02^{bc}	2.35±1.09^{c}	8.50±1.03^{ab}	4.68±1.15^{bc}	2.62±0.38^{c}
3-Methylundecane	1179	MS, RI	1002-43-3	$C_{12}H_{26}$	NE	ND	4.21±0.15^{b}	5.40±0.02^{a}	2.64±0.34^{c}	5.08±0.35^{ab}	2.07±0.42^{c}
Dodecane	1212	MS, RI	112-40-3	$C_{12}H_{26}$	Popcorn-like aroma	0.77±0.14^{b}	8.13±1.04^{a}	9.18±0.62^{a}	9.90±1.35^{a}	8.72±0.99^{a}	7.96±0.75^{a}
Tridecane	1300	MS	629-50-5	$C_{13}H_{28}$	NE	ND	4.01±0.28	ND	ND	2.69±0.15	2.87±0.48
2,6,10-Trimethyltridecane	1453	MS	3891-99-4	$C_{16}H_{34}$	NE	1.79±0.13^{b}	1.29^{b}	3.07±0.28^{ab}	4.61±0.96^{a}	2.71±0.57^{ab}	2.73±0.64^{ab}
Tetradecane	1411	MS, RI	629-59-4	$C_{14}H_{30}$	Mild waxy	1.00±0.02^{c}	2.49±0.21^{b}	2.70±0.28^{b}	5.82±0.72^{a}	3.01±0.04^{b}	2.64±0.27^{b}
**Heterocycles**											
2-Pentylfuran	1219	MS, RI	3777-69-3	$C_9H_{14}O$	Fruity,beany,metallic	0.44±0.02^{c}	1.22±0.07^{b}	2.51±0.24^{a}	2.69±0.18^{a}	3.01±0.33^{a}	0.54±0.06^{c}
5H-1-Pyrindine	2398	MS	270-91-7	C_8H_7N	NE	1.93±0.58	ND	ND	0.21±0.08	ND	ND
2-Pyrrolidinone	1978	MS	616-45-5	C_4H_7NO	NE	ND	ND	ND	0.34±0.04	0.25±0.02	
2-Acetylpyrrole	1933	MS, RI	1072-83-9	C_6H_7NO	Toasted, boullion	ND	ND	ND	ND	0.28±0.04	0.78±0.04
2-Isopropyl-4,5-dimethylthiazole	1431	MS	53498-30-9	$C_8H_{13}NS$	NE	ND	ND	ND	ND	ND	0.83±0.06
**Acids**											
Benzoic acid	2398	MS	65-85-0	$C_7H_6O_2$	Faint balsam, urine	ND	ND	ND	ND	0.24±0.10	ND
Acetic acid	1427	MS, RI	64-19-7	$C_2H_4O_2$	Vinegar-like	0.64±0.07^{d}	1.62±0.04^{b}	1.56±0.05^{b}	2.05±0.05^{a}	1.81±0.11^{ab}	1.03±0.18^{c}
Octanoic acid	2037	MS, RI	124-7-2	$C_8H_{16}O_2$	NE	ND	0.44±0.07	ND	ND	ND	ND
Decanoic acid	2252	MS, RI	334-48-5	$C_{10}H_{20}O_2$	Unpleasant rancid, fatty,	ND	ND	0.22±0.09^{a}	0.37±0.07^{a}	0.27±0.12^{a}	ND
Dodecanoic acid	2464	MS, RI	143-7-7	$C_{12}H_{24}O_2$	NE	0.32±0.04	0.45±0.13	0.53±0.13	0.29±0.05	ND	ND
Tetradecanoic acid	2675	MS	544-63-8	$C_{14}H_{28}O_2$	Waxy, fatty	0.88±0.04^{ab}	1.11±0.18^{a}	ND	ND	0.64±0.04^{bc}	0.37±0.10^{c}

Pentadecylic acid	2780	MS	1002-84-2	$C_{15}H_{30}O_2$	Waxy	0.83±0.02^{b}	ND	1.27±0.06^{a}	ND	0.22±0.04^{d}	0.42±0.01^{c}
**Amines**											
Trimethylamine	656	MS, RI	75-50-3	C_3H_9N	Fishy, ammonia	9.21±0.62^{d}	57.79±0.19^{c}	88.37±3.99^{b}	56.53±6.12^{c}	80.44±7.85^{b}	112.37±8.45^{a}
3-Methyl-butanamide	1877	MS	541-46-8	$C_5H_{11}NO$	NE	ND	ND	ND	0.65±0.15	ND	ND
Succinimide	2411	MS	123-56-8	$C_4H_5NO_2$	NE	0.84±0.06^{a}	ND	0.37±0.16^{b}	0.24±0.05^{b}	0.14±0.04^{b}	ND
Acetamide	1719	MS	60-35-5	C_2H_5NO	Bland	ND	ND	ND	ND	0.81±0.11	0.15±0.03

[1] RI:Linear Retention Index, calculates the retention index of a compound on an InertCap® Pure-WAX column based on the retention time of n-alkanes (C6-C28) on the instrument.

[2] Identification approach. MS: mass spectrum; RI: retention index.

[3] Reference reports [1,2] and web databases(http://www.odour.org.uk, http://www.flavornet.org); NE: not estimated.

[4] The concentration of volatile compounds is expressed as the mean ± standard deviation (Mean ± SD) of triplicate samples; Different letters within a row indicate significant differences (p < 0.05)

[5] Different letters in the same row indicate significant differences between samples (P < 0.05); ND:not detected.

[6] BS: blanching shrimp; VFDS: vacuum freeze-dried shrimp; VDS: vacuum-dried shrimp; HPDS: heat pump-dried shrimp; HADS: hot air-dried shrimp; MVDS: microwave vacuum-dried shrimp.

1. Yu, J.; Lu, K.; Zi, W.J.; Yang, X.H.; Xie, W.C. Characterization of aroma profiles and aroma-active compounds in high-salt and low-salt shrimp paste by molecular sensory science. *Food Biosci*. **2022**, *45*, 101470.
2. Zhu, Y.; Xiaoting Chen, X.T.; Pan, N.; Liu, S.J.; Su, Y.C.; Xiao, M.T.; Shi, W.Z.; Liu, Z.Y. The Effects of Five Different Drying Methods on the Quality of Semi-Dried Takifugu Obscurus Fillets. *LWT Food Sci. Techno*l. **2022**, *161*, 113340.
